# Microcystic/reticular schwannoma of the esophagus: the first case report and a diagnostic pitfall

**DOI:** 10.1186/s12876-014-0193-y

**Published:** 2014-11-18

**Authors:** Mi Jin Gu, Joon Hyuk Choi

**Affiliations:** Department of Pathology, Yeungnam University College of Medicine, Daegu, Republic of Korea

**Keywords:** Schwannoma, Microcystic, Reticular, Esophagus

## Abstract

**Background:**

Microcystic/reticular schwannoma is a recently described, rare, distinctive histological variant of schwannoma with a predilection for the gastrointestinal tract (GIT). The authors experienced the first case of a microcystic/reticular schwannoma occurring in the esophagus.

**Case presentation:**

A 39-year-old male presented for an obstructive sensation during swallowing of several months duration. Endoscopy revealed a bulging mass with intact mucosa at 30 cm from incisors in the esophagus. The mass was excised and gross examination showed it was a well circumscribed, unencapsulated nodule, measuring 3.5×3.2×1.2 cm. On microscopic examination, the tumor showed a vague multinodular appearance with a pushing border and tumor cells arranged in a microcystic and reticular growth pattern with anastomosing and intersecting strands of spindle cells in a myxoid or collagenous/hyalinized stroma. Tumor cells showed diffuse nuclear and cytoplasmic positivity for S100.

**Conclusions:**

The authors report the first case of microcystic/reticular schwannoma of the esophagus. Microcystic/reticular schwannoma is a distinctive histological variant of schwannoma with a benign clinical course. However, its histological findings are non-specific and may cause diagnostic difficulties. Awareness of this uncommon neoplasm with distinct histologic features is essential to prevent misdiagnosis.

## Background

Schwannomas are benign mesenchymal neoplasm and usually arise in the subcutaneous tissues of the distal extremities or in the head and neck region [1]. Gastrointestinal tract (GIT) schwannomas are rare and have histological and immunophenotypical features similar to those of non-GIT schwannomas. Several morphologic variants of schwannoma are recognized, that is, conventional, cellular, microcystic/reticular, plexiform, and melanotic schwannoma [1,2]. Microcystic/reticular schwannoma is a recently described, rare, distinctive histological variant of schwannoma with a predilection for the GIT. Twelve cases of microcystic/reticular schwannoma of the GIT have been reported in the English literature [1-4]. Herein, we report the first case of microcystic/reticular schwannoma of the esophagus and include a review of the literature.

## Case presentation

**Figure 1:**
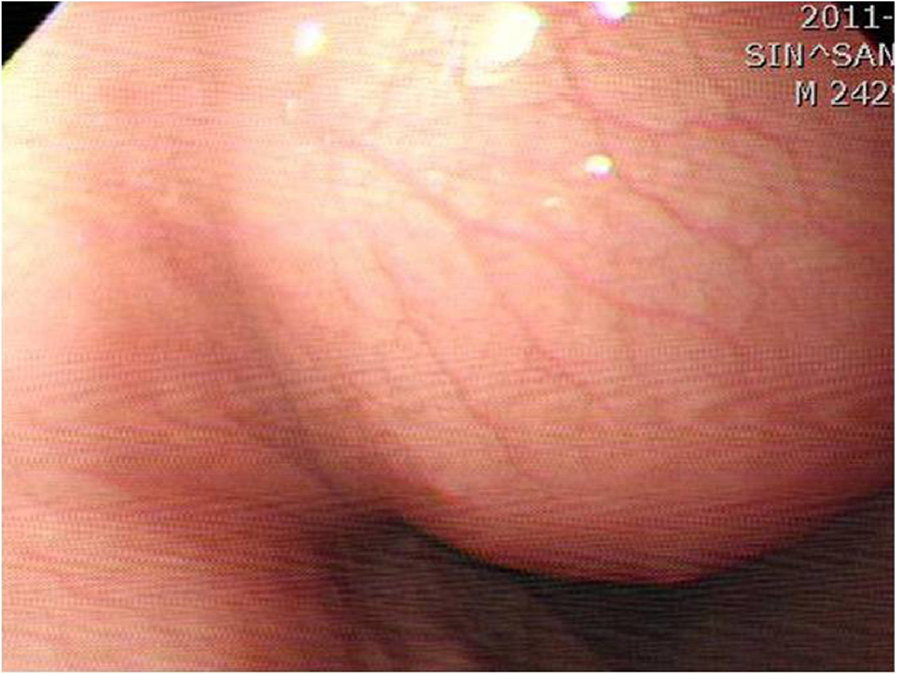
**Endoscopic examination revealed a bulging mass with intact mucosa in the mid esophagus.**

**Figure 2:**
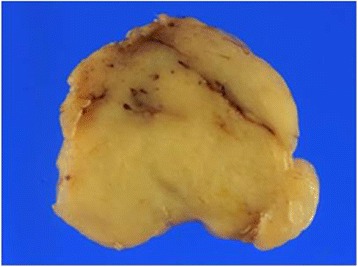
**Gross findings.** Cut surfaces showed whitish yellow, homogeneously solid, rubbery tissue with a myxoid appearance.

**Figure 3:**
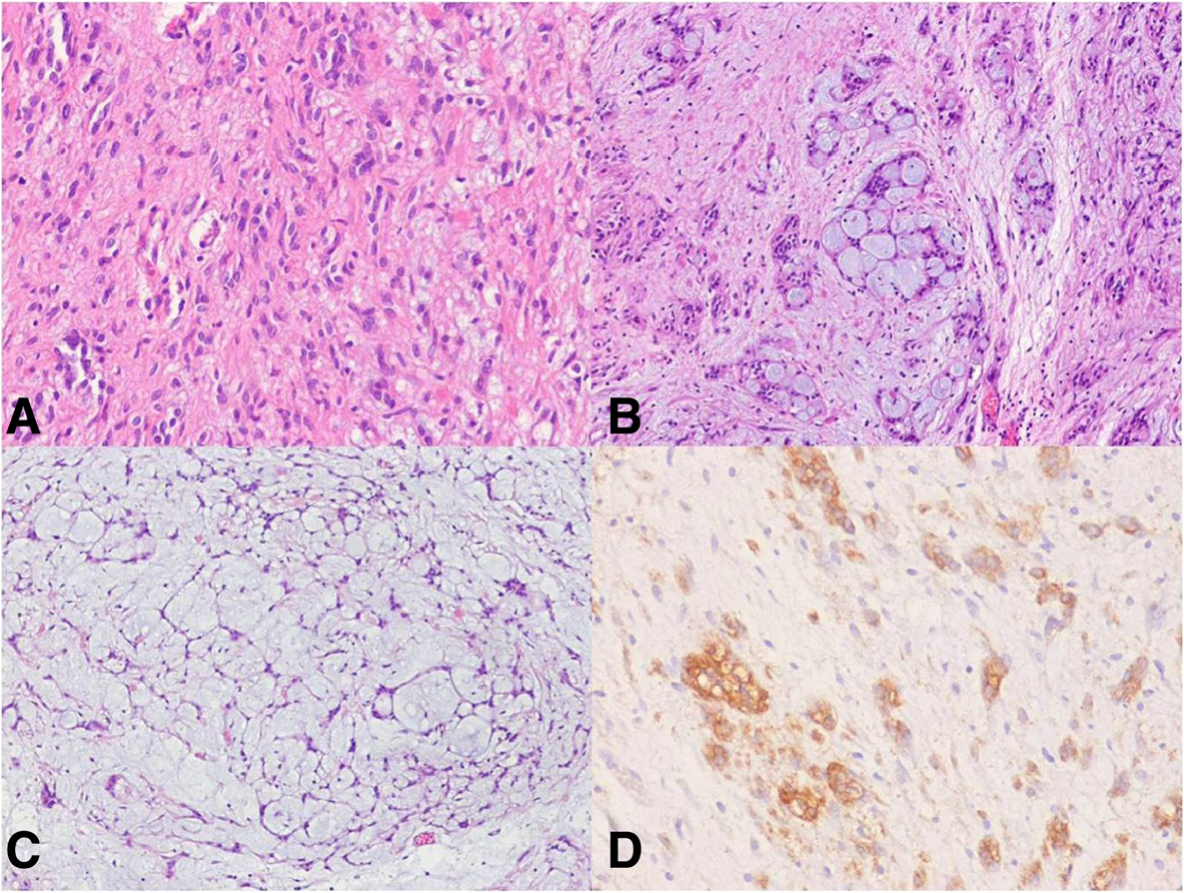
**Microscopic findings. A)** Elongate slender or plump spindle cells with oval nuclei and eosinophilic cytoplasm (original magnification 100×), **B**-**C)** Photograph showing a microcystic and reticular growth pattern with anastomosing and intersecting strands of spindle cells in myxoid or collagenous/hyalinized stroma (original magnification 100×). **D)** Immunohistochemistry showed strong positivity for S100 (original magnification 100×).

A 39-year-old male presented for an obstructive sensation during swallowing of several months duration. During endoscopy, a bulging mass with intact mucosa was observed in the esophagus at 30 cm from incisors (Figure 1). Endoscopic ultrasonograpy revealed a homogeneously hypo- to iso-echoic mass measuring 2.3 cm in size in the submucosal layer. Mass excision via video-assisted thoracoscopic surgery (VATS) was performed. The resected mass was a well circumscribed, unencapsulated nodule, measuring 3.5×3.2×1.2 cm. Cut sections showed whitish yellow, homogeneously solid, rubbery tissue with a myxoid appearance (Figure 2). On microscopic examination, the tumor showed a vague multinodular appearance with a pushing border and was composed of elongate slender or plump spindle cells with oval nuclei and eosinophilic cytoplasm (Figure 3A). Its cells were arranged in a microcystic, reticular growth pattern with anastomosing and intersecting strands of spindle cells in a myxoid or collagenous/hyalinized stroma (Figure 3B,C). No cytologic atypia, necrosis, or mitosis was observed. In a collagenous/hyalinized area, hypocellular and hypercellular areas with vague nuclear palisading were observed, but no well-developed Verocay body was identified. Sparse perivascular lymphoplasma cell infiltrations were observed. However, prominent nodular lymphoplasmacytic infiltrates, thick and hyalinized vessels, hemorrhage with hemosiderin deposition, calcification, and cyst formation was absent, and the mitotic count did not exceed 1/50HPF. Tumor cells showed diffuse nuclear and cytoplasmic positivity for S100 (Figure 3D), but were negative for CD117, CD34, smooth muscle actin, synaptophysin, chromogranin, GFAP, and AE1/AE3. Throughout a follow-up of 27 months, the patient remained well without tumor recurrence or metastasis.

## Discussion

Schwannomas account for 2 ~ 7% of mesenchymal GIT neoplasms and the stomach is the most common site [1]. In fact, More than 80% of benign esophageal tumors are leiomyomas [5]. Esophageal schwannomas are exceedingly rare, benign, and do not recur after complete excision [6]. Schwannoma has several morphologic variants, that is, conventional, cellular, microcystic/reticular, plexiform, and melanotic schwannoma. Schwannoma of the esophagus occur more frequently in women than in men and is usually encountered in the upper or mid esophagus. Recently, endoscopic ultrasonography-guided fine needle aspiration biopsy has been shown to be useful for preoperative diagnosis [5].

To the best of our knowledge, this is the first report of a microcystic/reticular schwannoma in the esophagus. However, 12 cases of reticular and microcystic schwannoma in the GIT have been reported [1-3] (Table 1); three in the stomach, two in each of the sigmoid colon, jejunum, and cecum, and one in each of the rectum, ascending colon, and small bowel. Nine of these 12 patients were female and three were male. Age at presentation ranged from 32 to 93 years (median 69 years), and tumors sizes ranged from 0.4 to 3.8 cm (mean 1.4 cm). Eleven of the 12 presented with a single mass and one with two masses. Neither recurrence nor metastasis was observed.

**Table 1:** **Clinicopathological features of microcystic/reticular schwannoma of the GIT**

**Case**	**Sex**	**Age (y)**	**Site**	**Size (cm)**	**Status of last follow-up**
1	F	73	Rectum	0.9	Died at 36 mo for colon cancer
2	F	72	Stomach	2.0	ANED 24 mo
3	M	68	Cecum	0.4	ANED 24 mo
4	F	93	Jejunum	1.6	ANED 7 mo
5	M	78	Small bowel	0.8	NA
6	F	63	Stomach	1.9	ANED 60 mo
7	F	70	Sigmoid colon	0.7	NA
8	F	32	Ascending colon	1.4	NA
9	F	67	Cecum	1.0	ANED 12 mo
10	F	67	Jejunum	2.2	ANED 12 mo
11	F	89	Stomach	3.8, 1.2	ANED 13 mo
12	M	61	Sigmoid colon	0.7	ANED 24 mo
Present case	M	39	Esophagus	3.5	ANED 27 mo

ANED, alive with no evidence of disease; NA, not available.

Our patient presented with a submucosal mass in the mid esophagus, which did not exhibit the conventional histological features of schwannoma, such as, nuclear palisading or Verocay body. Microscopically, the tumor showed a characteristic microcystic, reticular growth pattern with anastomosing and intersecting strands of spindle cells. Sparse perivascular lymphoplasma cell infiltrations were evident, but prominent nodular lymphoplasmacytic infiltrates were not observed. Liegl *et al*. also reported that lymphocytes cuffing with the germinal center were not observed in any of the case with a GIT location [1].

Therefore, variable entities should be considered to achieve a differential diagnosis, which includes gastrointestinal stromal tumor (GIST) with myxoid change, reticular variant of perineurioma, myoepithelial tumor, extraskeletal myxoid chondrosarcoma, low-grade fibromyxoid sarcoma (LGFMS), and ganglioneuroma with abundant myxoid stroma [1,2,7-10]. Gastrointestinal stromal tumors show a wide spectrum of histological features, but microcystic change is an unusual finding and immunoreactivity for CD117, CD34, and Dog-1 could aid the diagnosis of GIST. Histologically, reticular perineuriomas mimic microcystic/reticular schwannoma, but they usually occur in superficial soft tissue of the hands and feet and exhibit a characteristic immunophenotype, that is, positivity for EMA and negativity for S100 and GFAP. Histological findings of myoepithelial tumors with a reticular growth pattern may overlap with those of microcystic/reticular schwannoma, although their immunoreactivities for epithelial and myoepithelial markers aid their differentiation from microcystic/reticular schwannoma. Extraskeletal myxoid chondrosarcoma may show features similar to those of microcystic/reticular schwannoma because the tumor is composed of branching and anastomosing cords in chondromyxoid stroma; however, a distinct microcystic change is an unusual finding. Molecular testing for *EWS* gene translocation and focal or scattered S100 staining can help exclude microcystic/reticular schwannoma. Low-grade fibromyxoid sarcoma is characterized by spindle cell tumor with bland histological findings, but has a fully aggressive behavior and a high rate of recurrence and metastasis. This tumor is composed of bland spindled to stellate cells in myxoid and fibrotic stroma. However, there are often prominent curvilinear and branching vessels in the myxoid area and the tumor cells are negative for S-100 [8,9]. Ganglioneuroma usually presents as a large mass in the retroperitoneum or mediastinum that is composed of clusters of ganglion cells in a neuromatous stroma. Although ganglioneuroma with abundant myxoid stroma has been confused with microcystic/reticular schwannoma, tumor location and careful searching for ganglion cells can distinguish this entity from microcystic/reticular schwannoma [10].

## Conclusions

We describe the first case of a microcystic/reticular schwannoma occurring in the esophagus. Microcystic/reticular schwannoma is a distinctive histological variant of schwannoma with a benign clinical course. However, many pathologists and gastroenterologist are unlikely to be familiar with this entity, which could cause diagnostic difficulties. Awareness of this uncommon neoplasm with a distinct histology is essential to prevent misdiagnosis.

## Consents

A copy of the written informed consent provided by the patient prior to publication has been made available for review by the Editor-in-Chief of this journal.

## Abbreviations

GIT: Gastrointestinal tract
GIST: Gastrointestinal stromal tumor
